# Insufficient education is a key factor of incorrect interpretation of HIV self-test results by female sex workers in Democratic Republic of the Congo: A multicenter cross-sectional study

**DOI:** 10.1097/MD.0000000000014218

**Published:** 2019-02-08

**Authors:** Serge Tonen-Wolyec, Salomon Batina-Agasa, Jean De Dieu Longo, Ralph-Sydney Mboumba Bouassa, Laurent Bélec

**Affiliations:** a Ecole Doctorale Régionale d’Afrique Centrale en Infectiologie Tropicale, Franceville, Gabon; b Faculté de Médecine, Université de Bunia, Bunia; c Faculté de Médecine et de Pharmacie, Université de Kisangani, Kisangani, Democratic Republic of the Congo; d Centre National de Référence des Maladies Sexuellement Transmissibles et de la Thérapie Antirétrovirale, Bangui; e Unité de Recherches et d’Intervention sur les Maladies Sexuellement Transmissibles et le SIDA, Département de Santé Publique, Faculté des Sciences de la Santé de Bangui, Central African Republic; f Laboratoire de virologie, Hôpital Européen Georges Pompidou, and Université Paris Descartes, Paris, France.

**Keywords:** Democratic Republic of the Congo, female sex workers, French-speaking country, HIV, practicability, self-test, sub-Saharan Africa, WHO recommendations

## Abstract

We report on field interpretation of HIV self-testing among female sex workers (FSWs) and non-FSWs living in Democratic Republic of the Congo.

Two hundred and eight participants [76 (36.5%) FSWs; 132 (63.5%) non-FSWs] were enrolled in Kisangani and Bunia to evaluate their ability to read and interpret the results of a prototype HIV self-test (Exacto Test HIV, Biosynex, Strasbourg, France), according to WHO recommendations. Thirteen standardized tests (6 positive, 5 negative, 2 invalid) were proposed after successive random selection.

Two thousand seven hundred and four standardized tests (1248 positive, 1040 negative, 416 invalid) were interpreted; 2435 (90.1%) were correctly interpreted, whereas 269 (9.9%) were misinterpreted. In FSWs and non-FSWs, the test results were similarly correctly interpreted in 87.4% (864/988) and 91.6% (1571/1716), respectively. In multivariate logistic regression analysis, only the variable “educational level” remained strongly associated with the interpretation of positive, negative, and invalid HIV self-test results, but not the variables “commercial sex work” and “language chosen for instructions for use.” Incorrect interpretation was significantly higher in participants with insufficient educational level than in those with sufficient education level for positive (13.1% vs 2.6%; adjusted OR: 4.5), negative (22.3% vs 2.6%; adjusted OR: 5.3), and invalid test results (23.8% v 6.4%; adjusted OR: 3.6).

Incorrect interpretation of HIV self-test was as common in FSWs and non-FSWs. The lower was the educational level, the greater was the difficulty to interpret results correctly. These observations point that insufficient education level, rather than commercial sex work by itself, constitutes a key factor of incorrect interpretation of HIV self-test.

## 1 Introduction

HIV self-testing is a novel strategy that can enable early HIV testing, prevention interventions, and care in key populations at high risk of HIV infection, such as female sex workers (FSWs).^[1,2]^ However, the ability of individuals to make a correct interpretation of self-test results remains under debated.^[3–8]^ Recently, HIV self-test result's interpretation was found common among FSWs living in Kampala, Uganda.^[9]^

We report on field experience of the interpretation of HIV self-test results among FSWs and a general female population not practicing commercial sex work (non-FSWs) living in Democratic Republic of the Congo (DRC).

## 2 Methods

The CE IVD, lateral flow, immunochromatographic HIV rapid test [Exacto PRO Test HIV, Biosynex, Strasbourg, France] was adapted as CE IVD, finger-stick whole-blood HIV self-test (Exacto Test HIV, Biosynex).^[10]^ The test uses a combination of a specific antibody binding protein that is conjugated to colloidal gold dye particles and synthetic antigens (gp41, gp36) able to detect antibodies against HIV-1 or HIV-2 in whole-blood, serum or plasma, which are bound to the solid-phase membrane. The Exacto Test HIV fulfilled the following criteria:

(i) Capillary blood-based test detecting early HIV infection in a period as short as 4 to 8 weeks after exposure;
(ii) Sterile safety lancet;
(iii) Simplified blood sampling system;
(iv) Simplified buffer delivery system;
(v) Specimen presence control by blood deposit assessment and migration control band;
(vi) Results in 10 minutes.

A multicenter cross-sectional study on the interpretation of HIV self-test results of a capillary blood-based HIV self-test Exacto Test HIV (Biosynex, Strasbourg, France) having sensitivity and specificity estimated to be 99.99% and 99.90%,^[10]^ respectively, according to WHO recommendations,^[11]^ was carried out in FSWs and non-FSWs, with methodology previously described.^[7]^

In brief, volunteers were enrolled from five voluntary and counselling testing sites for HIV infection in facilities dedicated to serving key populations, including FSWs, in Kisangani and Bunia, DRC. All participants were volunteers seeking to know their HIV status. A structured questionnaire was used to obtain socio-demographic and sex behaviors data. FSWs were defined as having more than two sexual partners (other than their regular partner) during the last three months and reporting having received money or “gifts” in return of sexual relationships, as proposed.^[12]^

The inclusion criteria for the present study were as follows: female, age ≥ 18 years, self-professed ability to read the instructions for use of the HIV self-test, willingness to undergo HIV screening, and to give written informed consent to participate. The exclusion criteria were male gender, age < 18 years, illiteracy, and unwillingness to follow protocol instructions.

The simplified instructions for use of the Exacto Test HIV adapted for the Congolese general public were available in French, as well as Lingala and Swahili, which are vernacular languages widely used in the DRC.^[7]^

All participants were evaluated concerning the ability to read and interpret the results of the HIV self-test, according to WHO recommendations.^[11]^ Thus, in a private room supervised by an observer (all observers were physicians), 13 standardized tests (including 6 positive [comprising 3 tests with strong test bands and 3 tests with weak test bands], 5 negative and 2 invalid) were proposed to the participants for interpretation after successive random selection. These standardized tests were coded by numbers to determine the expected results (i.e., positive, negative, or invalid).

All data were entered into an Excel file and analyzed on SPSS 20.0 (Chicago, IL). The results were presented as a 95% confidence interval (CI) using the Wilson score bounds. The Pearson's *χ*^2^ test was used for comparison of the frequencies, while Fisher's exact test was used when the validity conditions of the latter test were not verified. The concordance between the results read by participants in connection with the expected results or as read by operators was estimated by Cohen's *κ* coefficient. The degree of agreement was determined as ranked by Landlis and Koch.^[13]^ To delineate and control possible confounders within the study variables and determine the independent predictors of the incorrect interpretation of the HIV self-test results, multivariable logistic regression analysis used significant variables from the bivariate analysis, which were arbitrarily taken as references for analyses, as previously described.^[7]^ The variable “educational level” included two categories according to the educational system of the DRC:^[14]^ “*insufficient*” [low (unschooled and primary schooled)] and “*sufficient*” [middle (college or technical school), and high (undergraduate degree and graduate degree)]. The *P*-value *<*.05 was considered as statistically significant. Finally, the sensitivity and specificity of the Exacto Test HIV as read by FSW and non-FSW were calculated according to the expected results. The positive predictive values (PPV) and negative predictive values (NPV) were calculated by taking into account the reported HIV prevalence in each population in DRC^[15]^. Note that the VPP and VPN of the Exacto Test HIV self-test in the hands of observers could be estimated to 99.2% and 100%, respectively, using Bayes’ formulae,^[16]^ and HIV prevalence of 1.2% in health care workers in DRC.^[15]^

Ethical was obtained from the Ethics Committee of the School of Public Health of the University of Kinshasa. Informed consent was obtained from all volunteers in writing.

## 3 Results

A total of 208 participants, including 76 (36.5%) FSWs and 132 (63.5%) non-FSWs, were enrolled. Note that 17 participants were excluded because they were minors (*n* = 14), and considered noncompliant (*n* = 3). The majority (71.6%, 149/208; 75.0% in FSWs and 69.7% in non-FSWs; *P* = .43) of participants were below 30 years of age, and self-reported the ability to read and write (77.9%, 162/208; 77.6% in FSWs and 78.0% in non-FSWs; *P* = .54). Insufficient educational level was observed in 57.7% (60.5% in FSWs and 56.1% in non-FSWs; *P* = .53). Only a minority had been previously tested for HIV (20.2%, 42/208; 18.4% in FSWs and 21.2% in non-FSWs; *P* = .23). The instructions for use were chosen in French by 30.3% (20.1% in FSWs and 35.6% in non-FSWs; *P* = .03), in Lingala by 30.8% (34.2% in FSWs and 28.8% in non-FSWs; *P* = .41), and in Swahili by 38.9% (44.7% in FSWs and 35.6% in non-FSWs; *P* = .19).

**Figure 1:**
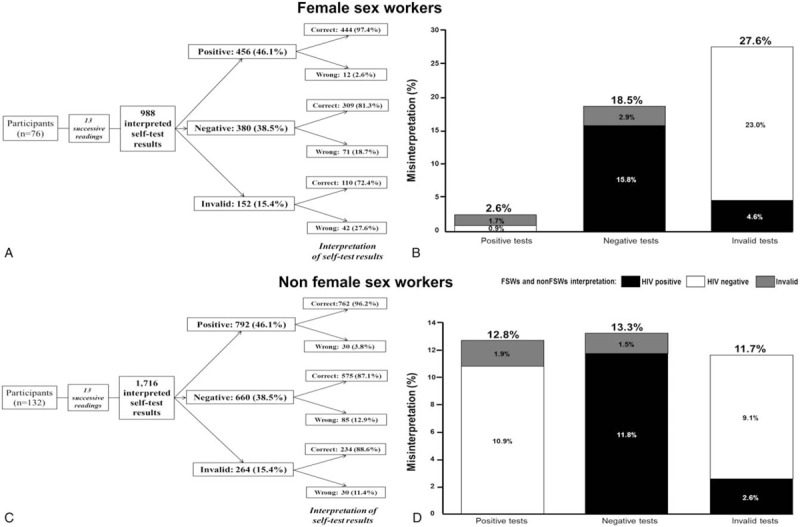
Flow charts showing the ability of participants to read and interpret (correctly or incorrectly) the 2704 standardized results of the Exacto Test HIV (Biosynex) obtained from successive random selection of a panel of 13 standardized tests, including 6 positive, 5 negative, and 2 invalid, including 988 by 76 female sew workers (FSWs) [**A**], and 1716 by 132 non-FSWs [**C**], and percentages of misinterpreted self-tests results by FSWs [**B**], and non-FSWs [**D**]. The heights of the vertical bars indicate the overall percentages of misinterpreted HIV self-test's results; the color-coded components of the bars indicated the type of misinterpretation: HIV-positive (black); HIV negative (white); invalid (gray).

This study evaluated the ability of participants to read and interpret the HIV self-test results from a panel of 13 standardized tests drawn successively (Fig. 1; Table 1). A total of 2704 standardized tests (including 1248 positive tests [comprising 624 tests with strong test bands and 624 positive tests with weak test bands], 1040 negative tests, 416 invalid tests) were read and interpreted by the 208 participants; 2435 (90.1%; 95%CI: 88.9–91.2) tests were correctly interpreted, whereas 269 (9.9%; 95%CI: 8.8–11.1) tests were misinterpreted. Misinterpretation occurred in 3.4% of positive tests (2.6% of positive tests with weak test bands comprising 2.1% incorrectly interpreted as negative and 0.5% as invalid, and in 0.8% of positive tests with strong test bands comprising 0.6% incorrectly interpreted as negative and 0.2% as invalid); in 14.9% of negative tests (including 12.9% incorrectly interpreted as positive and 2.0% as invalid); and in 17.3% of invalid tests (including 3.4% of tests falsely interpreted as positive and 13.9% as negative). The Cohen's κ coefficient between the interpretations of the HIV self-test results made by the participants and the expected results was 0.79, demonstrating excellent concordance.

**Table 1:**
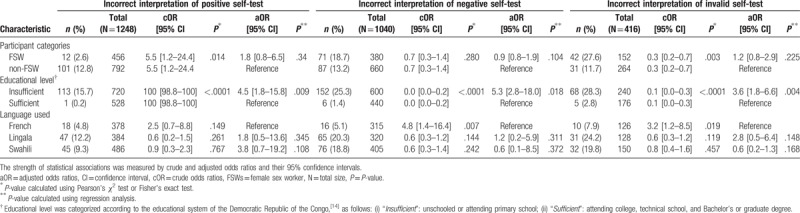
Bivariate and multivariate regression analysis of factors associated with incorrect interpretation of positive, negative, and invalid HIV self-test results among the 2704 results of the Exacto Test HIV (Biosynex) obtained from successive random selection of a panel of 13 standardized tests, including 6 positive, 4 negative, and 2 invalid by 208 adult female participants including 76 FSW and 132 non-FSW.

Among the subgroups of FSWs, the results were correctly interpreted in 87.4% (864/988) of cases. Among the positive, negative, and invalid self-tests, misinterpretation occurred in 2.6% (0.4% positive tests with strong test bands and 2.2% positive tests with weak test bands), 18.4%, and 27.4% of cases, respectively (*P* < .001). The Cohen's κ coefficient was 0.79. FSWs-interpreted HIV self-test sensitivity was 88.8% (95%CI: 86.5%–90.7%) and specificity was 86.1% (95%CI: 83.6%–88.3%), which is also the percentage of participants that correctly interpreted the positive (including tests with strong or weak test bands) and negative HIV self-test results, respectively. HIV prevalence among our study participants may be estimated at 7.6%,^[15]^ which translates into a FSW-interpreted HIV self-test positive predictive value (PPV) of 34.4% (95%CI: 31.3%–37.6%) and negative predictive value (NPV) of 98.9% (95%CI: 97.9%–99.4%).

Among the subgroups of non-FSWs, the results were correctly interpreted in 91.6% (1571/1716) of cases. Among the positive, negative, and invalid self-tests, misinterpretation occurred in 3.8% (0.9% positive tests with strong test bands and 2.9% positive tests with weak test bands), 12.9%, and 11.4% of cases, respectively (*P* < .001). The Cohen's κ coefficient was 0.79. Non-FSWs-interpreted HIV self-test sensitivity was 96.9% (95%CI: 95.9%–97.7%) and specificity was 87.3% (95%CI: 85.5%–88.9%). HIV prevalence among study Non-FSW participants may be considered similar to that of the general adult population in Bunia and Kisangani at 1.6%,^[15]^ which translates into a Non-FSW-interpreted HIV self-test PPV of 11.0% (95%CI: 9.5%–12.7%) and NPV of 99.9% (95%CI: 99.5%–100.0%).

**Figure 2:**
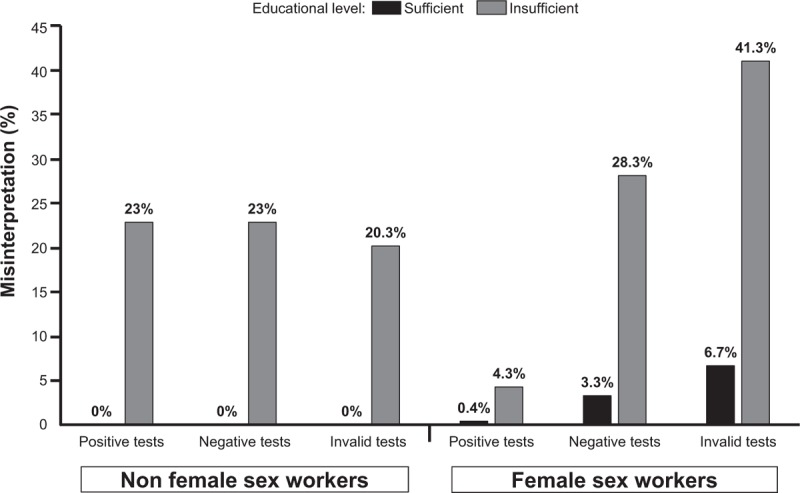
Inverse correlation between the education level and percentage of misinterpretation of results among FSW and non-FSW. Percentages of misinterpretation of test results were higher among FSWs with insufficient educational level as compared to those with sufficient educational level for positive (4.3% vs 0.4%, *P* = .005), negative (28.3% vs 3.3%, *P* < .001), and invalid (41.3% vs 6.7%, *P* < .001) test results. Percentages of misinterpretation of test results were higher among non-FSWs with insufficient educational level as compared to those with sufficient educational level for positive (23% vs 0%, *P* < .001), negative (23% vs 0%, *P* < .001), and invalid (20.3% vs 0%; *P* < .001) test results.

Finally, there was an inverse correlation between the education level and percentage of misinterpretation of results among FSW and non-FSW: FSWs with insufficient educational level incorrectly interpreted the self-test compared to those with sufficient educational level (4.3% vs 0.4%, *P* = .005 for positive tests, 28.3% vs 3.3%, *P* < .001 for negative tests, and 41.3% vs 6.7%, *P* < .001 for invalid tests). Among non-FSWs, no test (positive, negative or invalid) was incorrectly interpreted by participants with sufficient educational level (Fig. 2).

In bivariate analysis, the variables “commercial sex work,” “language used for the instructions for use,” and “educational level,” were factors associated with the interpretation of HIV self-test results (Table 1). In multivariate logistic regression analysis, the variables “commercial sex work” and “language used for the instructions for use” remained no more associated with the interpretation of HIV self-test results. The variable “educational level” remained associated with the interpretation of positive, negative, and invalid test results: Incorrect interpretation was higher in participants with insufficient education level than in those with sufficient education level for positive (13.1% vs 2.6%, *P* < .001; adjusted OR: 4.5 [95%CI: 1.8–15.8], *P* = .09), negative (22.3% vs 2.6%, *P* < .001; adjusted OR: 5.3 [95%CI: 2.8–18.0], *P* = .02), and invalid test results (23.8% vs 6.4%, *P* < .001; adjusted OR: 3.6 [95%CI: 1.8–6.6], *P* = .004).

## 4 Discussion

We herein report on our original and recent experience of interpretation of HIV self-test results among female adult volunteers living in Kisangani and Bunia in the DRC, using a finger-stick whole-blood HIV self-test (Exacto Test HIV, Biosynex). The participants were divided as FSWs and non-FSWs according to their statement of practicing commercial sex work. The majority of items in the group of FSWs were similarly observed in the control group of non-FSWs, except instructions for use in French more frequently used in Non FSWs than FSWs, assessing high comparability between groups. In substance, the unique characteristics of FSWs could not explain by themselves the lower HIV self-test performance measurements in our study, although FSWs may have lower levels of health knowledge and higher consumption of alcohol and other psycho-active substances.^[12]^ In contrast, incorrect interpretation of HIV self-test results was strongly associated with low educational levels. The lower was the educational level; the greater was the difficulty to interpret the test correctly, despite frequently used notice for instructions in vernacular language (s).

Our results confirm those reported by Ortblad and colleagues on FSWs in Uganda,^[9]^ in the DRC, a French-speaking African country, which has its own specific cultural, economic, and societal characteristics, possibly different to those of an English-speaking country. First, incorrect interpretation of HIV self-test results was common among FSWs living in Kisangani and Bunia, since around 1 out of 9 test results were misinterpreted by FSWs in our series. As in Ortblad's report,^[9]^ the FSWs-interpreted HIV self-test sensitivity and specificity measurements were below 90%, which is far lower than those measured in most of the previous HIV self-testing performance studies reported in sub-Saharan Africa (≥94% sensitivity and >98% specificity).^[1,3,4,17]^ Application of the observed sensitivity and specificity to a hypothetical group of 1000 FSWs living in the DRC with 20% of HIV prevalence^[18]^ would result in 22 HIV-positive FSWs being missed, and 28 HIV-negative FSWs being misidentified with a reactive test result. Furthermore, the calculated PPV in Bunia and Kisangani was very low (34.4%), indicating risk of misdiagnosis of two-third of tests interpreted as positive. Second, misinterpretation of HIV self-test occurred with each of the three test result possibilities, at seemingly high frequency (12.6%) in FSWs. The high frequency of misinterpretation in our study population differed greatly from that reported by Prazuck and colleagues in a non-trained general population living in France in which the vast majority of participants succeeded, with only 2.9% of the participants making errors, mostly when reading an invalid test.^[19]^ Thus, misinterpretation of inconclusive self-test results was the highest, in around 1 out 4 FSWs, as in Ortblad's report,^[9]^ but such results are rare in real-world settings.^[3]^ Incorrect HIV self-test results interpretation of negative tests in our series occurred in around 1 out of 5 FSWs, at similar frequency as in FSWs living in Uganda.^[9]^ For HIV positive test results, misinterpretation was more common with tests showing weak rather than strong bands, but at the difference to Ortblad's observations,^[9]^ the frequency of misinterpretation of HIV positive test (with weak or strong bands) was much lower in our series. These findings indicate that incorrect interpretation may occur for HIV oral fluid-based self-test as well as for capillary-based HIV self-test, and suggest that the ability to understand and correctly follow HIV self-test kit instructions for use may differ between both HIV self-tests. Finally, the development of an HIV self-test with the less as possible ambiguity to a low literacy public needs to be explored in sub-Saharan Africa.

In conclusion, our observations point that insufficient education level, rather than commercial sex work by itself, constitutes a key factor of incorrect interpretation of HIV self-test.

## Acknowledgments

The authors are grateful to the volunteers for their willingness to participate in the study. Thanks are also due to Biosynex, Strasbourg, France, for providing free the Exacto Test HIV self-tests for the study. We also thank the *Programme National de Lutte contre le SIDA*, Kinshasa, Democratic Republic of the Congo.

## Author contributions

S.T.-W., S.B.-A., and L.B. conceptualized the study. S.T.-W. oversaw data collection. S.T.-W., J.D.D.L. and R.-S.M.B. conducted the analysis. S.T.-W. and L.B. wrote the first draft. All authors edited the draft.

**Conceptualization:** Serge Tonen-Wolyec, Salomon Batina-Agasa, Laurent Bélec.

**Data curation:** Serge Tonen-Wolyec.

**Formal analysis:** Serge Tonen-Wolyec, Jean De Dieu Longo, Laurent Bélec.

**Investigation:** Serge Tonen-Wolyec.

**Methodology:** Serge Tonen-Wolyec, Salomon Batina-Agasa, Ralph-Sydney Mboumba Bouassa, Laurent Bélec.

**Supervision:** Salomon Batina-Agasa, Laurent Bélec.

**Validation:** Serge Tonen-Wolyec, Ralph-Sydney Mboumba Bouassa.

**Visualization:** Laurent Bélec.

**Writing – original draft:** Serge Tonen-Wolyec, Ralph-Sydney Mboumba Bouassa, Laurent Bélec.

**Writing – review & editing:** Serge Tonen-Wolyec, Laurent Bélec.

Serge Tonen-Wolyec orcid: 0000-0002-7734-7729.

## References

[1] Chanda MM Ortblad KF Mwale M HIV self-testing among female sex workers in Zambia: a cluster randomized controlled trial. PLoS Med 2017;14:e1002442. 29161260 10.1371/journal.pmed.1002442 PMC5697803

[2] Ortblad K Kibuuka Musoke D Ngabirano T Direct provision versus facility collection of HIV self-tests among female sex workers in Uganda: a cluster-randomized controlled health systems trial. PLoS Med 2017;14:e1002458. 29182634 10.1371/journal.pmed.1002458 PMC5705079

[3] Choko AT Desmond N Webb EL The uptake and accuracy of oral kits for HIV self-testing in high HIV prevalence setting: a cross-sectional feasibility study in Blantyre, Malawi. PLoS Med 2011;8:e1001102. 21990966 10.1371/journal.pmed.1001102 PMC3186813

[4] Figueroa C Johnson C Ford N Reliability of HIV rapid diagnostic tests for self-testing performed by self-testers compared to health-care workers: a systematic review and meta-analysis. Lancet HIV 2018;5:e277–90. 29703707 10.1016/S2352-3018(18)30044-4 PMC5986793

[5] Grésenguet G Longo JD Tonen-Wolyec S Acceptability and usability evaluation of finger-stick whole blood HIV self-test as an HIV screening tool adapted to the general public in the Central African Republic. Open AIDS J 2017;11:101–18. 29290887 10.2174/1874613601711010101 PMC5730956

[6] Longo JDD Saint-Calvaire Diemer H Tonen-Wolyec S HIV self-testing in Central Africa: stakes and challenges. Health Sci Dis 2018;19:19.

[7] Tonen-Wolyec S Batina-Agasa S Muwonga J Evaluation of the practicability and virological performance of finger-stick whole-blood HIV self-testing in French-speaking sub-Saharan Africa. PLoS One 2018;13:e0189475. 29320504 10.1371/journal.pone.0189475 PMC5761859

[8] Tonen-Wolyec S Mboup S Grésenguet G Insufficient education is a challenge for HIV self-testing. Lancet HIV 2018;5:e341. 10.1016/S2352-3018(18)30141-3 30052505

[9] Ortblad KF Musoke DK Ngabirano T Female sex workers often incorrectly interpret HIV self-test results in Uganda. J Acquir Immune Defic Syndr 2018;79:e42–5. 29847478 10.1097/QAI.0000000000001765 PMC6095458

[10] UNITAID. HIV rapid diagnostic tests for self-testing. 4th edition. Market and technology landscape. July 2018 Available at: https://unitaid.org/assets/HIV-Rapid-Diagnostic-Tests-for-Self-Testing_Landscape-Report_4th-edition_July-2018.pdf Accessed August 21, 2018.

[11] World Health Organization (WHO). WHO prequalification: Sample product dossier for an IVD intended for HIV self-testing. SIMU™ (self-test for HIV 12O working document. December 2015 Available at: http://www.who.int/sample_dos_self_testinghiv_for_public_c__comment_v1_pdf Accessed August 21, 2018.

[12] Longo JD Simaléko MM Ngbale R Spectrum of female commercial sex work in Bangui, Central African Republic. SAHARA J 2017;14:171–84. 29092678 10.1080/17290376.2017.1394907 PMC5678296

[13] Landlis JR Koch GG The measurement of observer agreement for categorical data. Biometrics 1977;33:159–74. 843571

[14] World Bank. Le système éducatif de la République Démocratique du Congo: Priorités et alternatives. January 2005 Available at: http://siteresources.worldbank.org/INTAFRREGTOPEDUCATION/Resources/444659-1210786813450/ED_CSR_DRCongo_fr.pdf Accessed August 21, 2018.

[15] République Démocratique du Congo. Ministère du Plan et Suivi de la Mise en œuvre de la Révolution de la Modernité (MPSMRM), Ministère de la Santé Publique (MSP) et ICF International, 2014. Enquête Démographique et de Santé en République Démocratique du Congo 2013–2014. Rockville, Maryland, USA: MPSMRM, MSP et ICF International. Available at: http://dhsprogram.com/FR300.pdf.2014 Accessed August 21, 2018.

[16] Jaeschke R Guyatt GH Sackett DL Users’ guides to the medical literature. III. How to use an article about a diagnostic test. B. What are the results and will they help me in caring for my patients? The Evidence-Based Medicine Working Group. JAMA 1994;271:703–7. 8309035 10.1001/jama.271.9.703

[17] Pant Pai N Sharma J Shivkumar S Supervised and unsupervised self-testing for HIV in high- and low-risk populations: a systematic review. PLoS Med 2013;10:e1001414. 23565066 10.1371/journal.pmed.1001414 PMC3614510

[18] Vandepitte JM Malele F Kivuvu DM HIV and other sexually transmitted infections among female sex workers in Kinshasa, Democratic Republic of Congo, in 2002. Sex Transm Dis 2007;34:203–8. 16878053 10.1097/01.olq.0000233743.57334.6a

[19] Prazuck T Karon S Gubavu C A finger-stick whole-blood HIV self-test as an HIV screening tool adapted to the general public. PLoS One 2016;11:e0146755. 26882229 10.1371/journal.pone.0146755 PMC4755564

